# Assessment of Contents and Health Impacts of Four Metals in Chongming Asparagus—Geographical and Seasonal Aspects

**DOI:** 10.3390/foods11050624

**Published:** 2022-02-22

**Authors:** Naifeng Xu, Hongxia Zhang, Jingze Jia, Hao Li, Zhaoxiang Zhu, Shuge Fu, Yuanfeng Wang

**Affiliations:** Institute of Engineering Food, College of Life Science, Shanghai Normal University, 100 Guilin Road, Xuhui District, Shanghai 200234, China; xunaifeng217@163.com (N.X.); 15996539654@163.com (H.Z.); jhappy100@163.com (J.J.); 1000496597@smail.shnu.edu.cn (H.L.); zzx1693199190@163.com (Z.Z.); fushuge@126.com (S.F.)

**Keywords:** metal content, health impacts, ICP-MS, asparagus, Chongming Island

## Abstract

In this paper, the contents of four typical metals (Pb, Cd, Hg, and As) in asparagus, water, and soil from Chongming Island were quantitatively determined by inductively coupled plasma mass spectrometry (ICP-MS). The contents of these metals in asparagus showed a common rule of Pb > As > Cd > Hg in different harvest seasons and regions. Significant seasonal differences were found in the contents by difference analysis, but no obvious regional differences were observed. Furthermore, the asparagus did not accumulate these four metals from the soil in Chongming Island by the assessment of bio-concentration factor. The asparagus was proved safe by the analysis of single-factor pollution index and Nemerow pollution index. Through combining the analysis of the above indexes and the geological accumulation index, we found that 51.62% of soil samples were mildly polluted by cadmium. The results of health risk analysis showed that the risk value of children was higher than that of adults under oral exposure, but the four metals in asparagus possessed no obvious risk to health. The above assessments illustrate that the daily consumption of asparagus in Chongming Island will not cause potential health impacts. It is of benefit to ensure the quality and economic interests of asparagus planting in Chongming Island through the investigation of this study.

## 1. Introduction

Many harmful substances have entered the ecosystem with the development of industrialization, modernization, and agricultural modernization progress [1,2,3]. Heavy metals and metalloids are inorganic contaminants in the environment. Due to their toxicity, persistence, and long-term accumulation [4], they have been recognized as one of global environmental problems [5,6]. These elements can enter soils in a variety of ways that can be generally divided into natural sources and anthropogenic sources [7]. In addition to natural geochemical processes, human activities can also cause them to enter soil. They will be enriched in the soil after long-term accumulation. Unlike organic pollutants, they are usually non-degradable [8] and have a long biological half-life, which is easy to accumulate in the food chain [9]. Therefore, the adverse effects of these elements on human health and on the environment have attracted considerable attention. The excessive accumulation of them may lead to deterioration of soil quality, affect the growth of crops, and even affect the ecological security of the region [6]. After entering the human body, these metals can combine with proteins and enzymes, then destroy the structure of proteins, inhibit the activity of enzymes, and finally affect human health through short-term or long-term exposure [10]. Studies have shown that excessive intake of these substances can damage the kidneys and other organs, and may also lead to cardiovascular diseases, neurological diseases, and even cancer [11,12].

Different metal elements, such as lead (Pb), cadmium (Cd), mercury (Hg), and arsenic (As), have different hazards to the human body. Lead is a heavy metal that has adverse effects on the central nervous system and may cause cognitive, physical, and behavioral impairment [13,14], being one that exists widely in nature. According to reports, long-term exposure to Cd can cause lung adenocarcinoma, lung cancer, renal dysfunction, and fractures [15]. It is reported that cadmium is a priority metal that causes ecological risks in the soil of Beijing, Anshan, and other places in recent years in China [16,17,18]. Long-term exposure to mercury can lead to the accumulation in adipose tissue and damage in human central nervous system [19]. Arsenic, a metalloid element with high content in the environment [20], has been linked to skin lesions, lung cancer, and other diseases [21,22]. Due to the harmfulness of these elements, it is necessary to monitor and evaluate their concentration in vegetables and the environment. Their standards of limits in vegetables vary from country to country. The limit standard of Pb and Cd in vegetables by Codex Alimentarius Commission (CAC) is 0.1 mg/kg and 0.05 mg/kg, respectively. The Eurasian Economic Union (EAEU) standard stipulate that the limits for Pb, Cd, Hg, and As in vegetables are 0.5 mg/kg, 0.03 mg/kg, 0.02 mg/kg, and 0.02 mg/kg, respectively, while they are 0.1 mg/kg, 0.05 mg/kg, 0.01 mg/kg, and 0.5 mg/kg, respectively, in Chinese national standard (GB 2762-2017).

Chongming Island, located in the north of Shanghai, China, takes vegetable cultivation as its main industry and supplies one-seventh of the vegetables in Shanghai. The metal source in Chongming Island is mainly fertilizer and traffic emissions due to no industrial zone being near the agricultural planting area and having a clean water source nearby. Asparagus is a characteristic agricultural product of Chongming Island. Because of the high phenol content in its edible parts, asparagus is one of the vegetables with strong antioxidant capacity and is beneficial to human health [23,24,25]. Studies have found that it can reduce the risk of cardiovascular and cerebrovascular diseases such as cancer and high blood pressure [26,27,28,29,30]. Asparagus is a vegetable in daily diet and has potential to develop functional products [31]. Therefore, understanding the metal contents, pollution levels, and health risks in asparagus and soil is of great significance to ensure the ecological safety of soil, the planting and consumption safety of asparagus, and ultimately the economic benefits of asparagus in Chongming area.

This study aimed to evaluate the pollution of metals in asparagus, soils, and water in Chongming Island, as well as possible risks to the human health through several ecological and health risk assessment methods. The bio-concentration factor is used to assess the ability of metals to migrate from soil to asparagus. With the research of Kowalska et al. [32] on pollution indicators, the soil pollution was evaluated by single-factor pollution index (SFPI), Nemerow pollution index (NPI), and geo-accumulation index (I_geo_). The risk exposure model recommended by the United States Environmental Protection Agency (USEPA) was employed to quantify health risks of asparagus consumption. The main purpose of this study was to (1) evaluate the metal pollution in asparagus, soil, and water in Chongming Island; (2) analyze the regional and seasonal differences of metal contents; and (3) analyze the potential health risks to people eating asparagus.

## 2. Materials and Methods

### 2.1. Description of the Study Area

Chongming Island is located at the mouth of the Yangtze River in the northeast of Shanghai (E 121°37′42″~121°47′41″, N 31°29′37″~31°34′29″). It is one of the largest alluvial islands in the world, formed by silt and soil accumulated over the years from the upper reaches of the Yangtze River [33]. Asparagus was introduced in Chongming Island in the 1880s and it has become a featured agricultural product after decades of development. The area of asparagus cultivation in Chongming accounts for more than 80% of total area of asparagus production in Shanghai, and it has become one of the important pillar industries for local agricultural efficiency and farmers’ income.

### 2.2. Sample Collection

**Figure 1:**
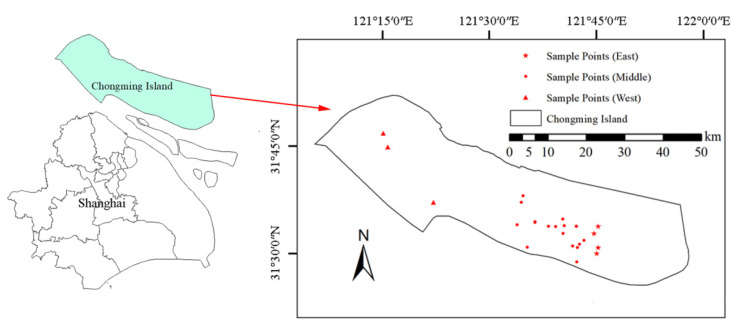
The study area and the distribution of sampling points.

We divided Chongming Island into three regions (east, middle, and west) according to the asparagus planting areas, and representative cooperatives were selected for sampling in each region. The sampling points are shown in the Figure 1. The eastern part of Chongming Island is the Dongtan Wetland [34], the western part has fewer asparagus planting areas, while the central part is the representative area of asparagus planting, and thus more sampling points were set up in the central part. We collected asparagus and the soil at the corresponding location to analyze the enrichment regularity of the elements under investigation. Sampling was carried out in three different harvest seasons of spring (in March 2021), summer (in July 2020), and autumn (in October 2019) to determine the seasonal variation of element content. The average temperature for these three months was 6–15 °C, 27–34 °C, and 15–23 °C. The soil samples were collected with reference to Chinese national standards (GB/T 36197-2018), and water samples were taken from the irrigation water source of the asparagus planting base. We collected a total of 238 samples at 14 sampling points in three time points, namely, 95 of asparagus, 95 of soil, and 48 of water, separately.

### 2.3. Sample Processing and Analysis

The collected asparagus samples were washed and dried, then ground with a grinder for later use. Referring to the sample processing in the national standard (GB 5009.268-2016), we accurately weighed 0.5 g of asparagus powder into a 50 mL centrifuge tube, and we added 2 mL of concentrated nitric acid and 1 mL of hydrogen peroxide for digestion, then put it in a graphite furnace at 120 °C for 2 h. Finally, ultrapure water was added to make the volume up to 25 mL. The soil samples were mixed and dried. After the removal of foreign bodies such as animal and plant residues, the soil samples were ground into powder by grinder and put through a 100-mesh (0.149 mm) nylon sieve. We accurately weighed 0.2 g of soil powder into a 50 mL centrifuge tube and added 1 mL of nitric acid, 1 mL of hydrochloric acid, and 2 mL of hydrofluoric acid for digestion to determine the concentration of lead and cadmium (GB/T 17141-1997). We added 3 mL hydrochloric acid and 1 mL nitric acid for digestion to determine the concentration of mercury and arsenic (GB/T 22105-2008). Other operations were the same as the asparagus sample. The background values of lead, cadmium, mercury, and arsenic in Shanghai soil we referred were 25.45 mg/kg, 0.125 mg/kg, 0.1 mg/kg, and 9.1 mg/kg, respectively. We ground up asparagus and soil by a high rotated speed disintegrator with main parameters of FW100, 24,000 rpm, rated power of 0.46 kw, etc., supplied by Tianjin Taisite Instrument Co., Ltd. (Tianjin, China). The processing method of the above samples was improved on the basis of national standard. The water sample was filtered with a 0.22 μm membrane (PES) and used in reserve. A total of 10 mL of filtered water sample was taken, and 0.2 mL nitric acid was added and mixed evenly. The concentrations of lead, cadmium, mercury, and arsenic in all samples were quantitatively determined by ICP-MS (Agilent 7900) in the Analysis and Testing Center of Shanghai Normal University. We weighed 10.0 g of soil into a 50 mL centrifuge tube, added 25 mL of water, stirred vigorously for 5 min, and let it stand for 2 h before measuring with a pH meter. The purity grade of all the chemicals we used was guaranteed reagent (GR), and the suppliers of these was Sinopharm Chemical Reagent Co., Ltd. (Shanghai, China).

### 2.4. Metal Pollution and Risk Assessment Methods

#### 2.4.1. Bio-Concentration Factors

The bio-concentration factors (BCF) can be used to study whether plants can absorb and enrich metals from the soil into plants. The calculation formula of BCF is as follows:

$$\mathrm{BCF}=\frac{c_{\mathrm{plant}}}{c_{\mathrm{soil}}}$$

where $c_{\mathrm{plant}}$ is the concentration of a metal in asparagus (mg/kg), and $c_{\mathrm{soil}}$ is the concentration of the metal in the soil where the asparagus grows (mg/kg).

#### 2.4.2. Pollution Index

The single-factor pollution index is used to evaluate the pollution degree of a single metal in each sample, and the calculation formula is as follows:

$$\mathrm{SFPI}=\frac{c_{i}}{s_{i}}$$

where $c_{i}$ is the concentration of a metal element in asparagus (mg/kg), and $s_{i}$ is the limit standard value of the metal in soil where the asparagus grows (mg/kg). Here, we adopted the limit value in the Chinese national standard (GB 2762-2017 for asparagus, GB 15618-2018 for soil).

The calculation formula of Nemerow index (NPI) is as follows:

$$\mathrm{NPI}=\sqrt{\frac{{\left({\mathrm{SFPI}}_{\max}\right)}^{2}+{\left({\mathrm{SFPI}}_{\mathrm{ave}}\right)}^{2}}{2}}$$

where ${\mathrm{SFPI}}_{\max}$ is the maximum value of SFPI of each sampling point, and ${\mathrm{SFPI}}_{\mathrm{ave}}$ is the arithmetic mean of the SFPIs of all metals in each sampling point. The classification criteria of these pollution indexes are shown in Table 1.

#### 2.4.3. Geo-Accumulation Index

The geo-accumulation index (I_geo_) method is used to comprehensively analyze and evaluate the metal pollution in Chongming area, and it is one of the evaluation methods widely used to evaluate metals in soils currently [35].

The calculation formula of I_geo_ is as follows:

$$I_{\mathrm{geo}}=\log_{2}^{\frac{c_{i}}{K\times B_{i}}}$$

where $c_{i}$ (mg/kg) refers to the measured concentration of metal i and $B_{i}$ refers to the environmental background value of metal i; the background value of the soil in Shanghai was adopted in this study. K refers to the coefficient used after considering the possible changes in the background value caused by rock differences, which is usually 1.5 [36]. The classification criteria of the I_geo_ are shown in Table 2.

#### 2.4.4. Human Health Risk Assessment Methods

At present, the human health risk assessment model proposed by the National Environmental Protection Agency (USEPA) is the most commonly used to assess the impact of metals in food on human health. People are generally exposed to metals in three ways: oral intake, oral and nasal inhalation, and skin contact. The main way for people to be exposed to the metals contained in asparagus is oral intake, and therefore the health risk of humans is assessed by this way.

The calculation formula for the exposure dose of oral exposure pathways is as follows:

$$\mathrm{ADI}=\frac{c\times E_{f}\times E_{D}\times E_{\mathrm{IR}}}{W_{\mathrm{AB}}\times T_{A}}\;$$

where ADI is the daily exposure dose, c is the metal content in asparagus, $E_{f}$ is the exposure frequency, $E_{D}$ is the exposure time, $E_{\mathrm{IR}}$ is the ingestion rate, $W_{\mathrm{AB}}$ is the weight of the child or adult, and $T_{A}$ is the average time of exposure to non-carcinogenic or carcinogenic metals.

Health risks can be divided into non-carcinogenic or carcinogenic risks. The non-carcinogenic risk is assessed by calculating the hazard quotient (HQ) value [37]. It is calculated as follows:

$$\mathrm{HQ}=\frac{\mathrm{ADI}}{R_{f}D}$$

where $R_{f}D$ refers to reference dose of metals.

When HQ < 1, it is considered that metal pollution has no significant health threat to the human body. When HQ > 1, it is considered that there is a health risk, and the larger the HQ value is, the greater the impact.

HI refers to the compound risk of multiple metals. Its judgment standard is the same as HQ, and its calculation formula is as follows:

HI=∑HQ

The formula for calculating the carcinogenic health risk index is as follows:

CR=ADI×SF

where CR refers to the carcinogenic health risk index of metals in the exposure pathway, and SF is the carcinogenic slope factor (Cd: 6.1, As: 1.5) [38]. When CR < 1.0 × 10^−6^, it means that the metal has no carcinogenic risk; when 1.0 × 10^−6^ < CR < 1.0 × 10^−4^, it means that the carcinogenic risk is acceptable; when CR > 1.0 × 10^−4^, it means the metal has carcinogenic risk.

At present, there is no completely unified standard for the value of each parameter in the formula for reference. The names and values of each parameter in this article are shown in Table 3.

### 2.5. Data Processing and Analysis

Statistical analysis of the data was performed using Origin Lab 2020, SPSS 22.0, and Microsoft Excel 2016. Charts are drawn by using Arcmap 10.6 (ESRI, Redlands, CA, USA) and Graphpad Prism 7 software (GraphPad Software Inc., San Diego, CA, USA).

## 3. Results and Discussion

### 3.1. Contents of Metals

**Figure 2:**
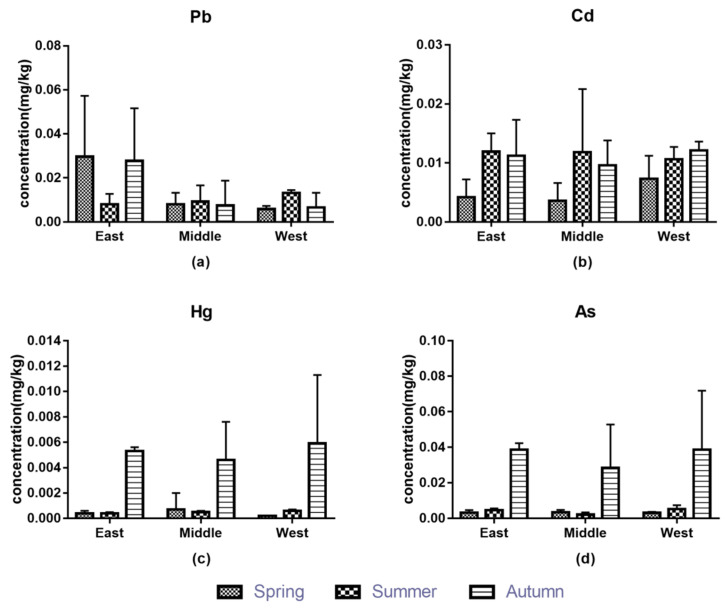
The contents of metals in asparagus: (**a**) Pb, (**b**) Cd, (**c**) Hg, (**d**) As.

Determined by ICP-MS, the contents of four metals in asparagus did not exceed Chinese national standard (GB 2762-2017), and their contents varied significantly in different seasons under this standard (Figure 2). The content of cadmium in summer or autumn was about three times that in spring. In autumn, lead content was about six times, while the content of mercury and arsenic were about 10 times in spring or summer. The growth of asparagus in spring was dominated by tender stems, and the continuous growth of stems and leaves in summer and autumn as well as the absorption of metals from the air may lead to the increasing contents [43]. Concerning the contents of metals, the lowest in every season was mercury, and the highest was lead in spring, cadmium in summer, and arsenic in autumn. The low mercury content in vegetables may be due to its strong combination with soil minerals and organic matter [44]. Judging from the regional distribution characteristics of metals, we found that there were relatively small regional differences of contents in asparagus in the same season. Research by Kirkillis [45] showed that the metal content of plants grown in industrial areas was higher than that in unpolluted non-industrial areas. Therefore, it is necessary to pay attention to the distance between asparagus planting areas and industrial areas. Our study found that the average content of lead in eastern asparagus was the highest at 0.0226 mg/kg, and arsenic in western was the highest at 0.0126 mg/kg, while in the three regions, the average content of cadmium and mercury was about 0.0085 mg/kg and 0.0015 mg/kg, respectively. Combining the seasonal and regional analysis of the distribution characteristics of these metals, we found that the lead content in the same area changed noticeably with the seasons—cadmium content was highest in autumn in the western region and highest in summer in the eastern and central regions, and the differences were distinct in spring and summer (*p* < 0.05); the contents of mercury and arsenic were the highest in autumn, and there were significant seasonal differences (*p* < 0.05). Interestingly, cadmium and arsenic contents in asparagus from Chongming area were higher than those from southern Italy [46], while lead and cadmium contents were lower compared to the contents from Chongqing market [47].

**Figure 3:**
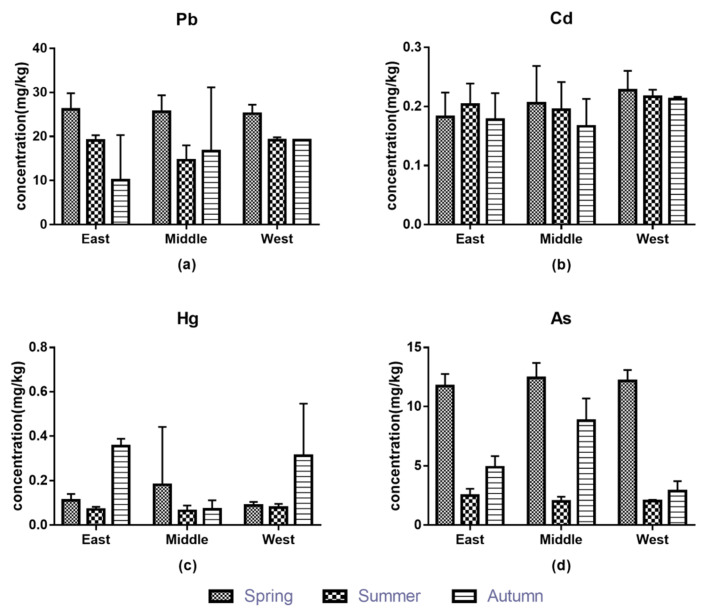
The contents of metals in soil: (**a**) Pb, (**b**) Cd, (**c**) Hg, (**d**) As.

The mean values of Pb and As (20.2520 mg/L for Pb and 7.9159 mg/L for As) in soil were lower than the Shanghai soil background values that were used as a reference system, while the mean values of Cd and Hg (0.1954 mg/L for Cd and 0.1263 mg/L for Hg) were higher than the background values [48]. In Figure 3, the contents of four elements in soil showed similar rules in three sampling seasons: Pb > As > Cd > Hg. In the three harvest seasons, the highest cadmium content was found in the west, which may have been due to a higher background value than that in the eastern and central regions. In the three regions, the highest contents of lead and arsenic were in spring, and the change of arsenic content was spring > autumn > summer; cadmium content in spring was highest in the central and western regions, and the east region was summer. Mercury content in autumn was the highest in the east and west, and for the middle region was spring. Comprehensive analysis of metal contents in soil in different regions showed that lead and cadmium contents were highest (22.1828 mg/kg and 0.2210 mg/kg, respectively) in the western region, mercury content was highest (0.1549 mg/kg) in the eastern region, and arsenic content was highest (8.0947 mg/kg) in the central region. Spring was the season in which the average contents of the four metals in the soil were highest, and the lowest was in summer. This may have been due to the conservation and fertilization of the asparagus planting land in winter, which led to the high content of metals in the soil. The results of difference analysis showed that significant differences were found in arsenic content in different seasons, and significant differences of lead content were found in spring and autumn, as well as those of mercury content being in summer and autumn (*p* < 0.05). The reason may have been the difference in absorption and utilization of each type of metal by plants in seasons. In addition, no correlation was found among metals in crops and soil (*p* < 0.05) in previous studies [49,50,51,52].

**Figure 4:**
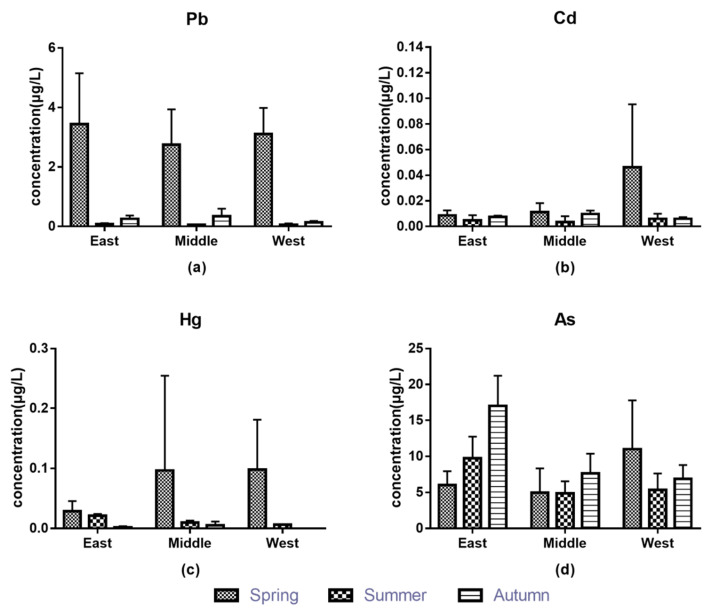
The contents of metals in water: (**a**) Pb, (**b**) Cd, (**c**) Hg, (**d**) As.

Located at the mouth of the Yangtze River, the western water source of Chongming Island is fresh water, while its eastern part is close to the sea and the source is mostly sea water. There are differences in the types and contents of metal ions in different water sources, and therefore four metal contents in irrigation water in three regions were analyzed, as shown in Figure 4. The contents of these metals in the three irrigation water samples were low (lead < 0.007 mg/L, cadmium < 0.0001 mg/L, mercury < 0.0005 mg/L, arsenic < 0.022 mg/L), being far below Chinese national standard limits. These metals in the irrigation water had no effect on the metal contents in soil and plants of the planting area, which is consistent with the results of Gan’s research [53]: there was no obvious correlation between metals in vegetables and irrigated water. Except for arsenic, the contents of metals in irrigation water were the highest in spring, and there were few differences between summer and autumn. The maximum arsenic content was in autumn, and the differences between spring and summer were small. In the three regions, the variation pattern of lead content was spring > autumn > summer, and the mercury content was spring > summer > autumn. The change rule of metal contents in water in each region was arsenic > lead > mercury > cadmium. Lead and arsenic contents were highest in the east, while cadmium and mercury contents were highest in the west.

A comprehensive comparison of the distribution characteristics and variation rules of metal contents in soil and asparagus may well explain the reasons for the relatively high quality and price of asparagus in spring.

### 3.2. Bio-Concentration Factor

The ability of plants to accumulate metals is described by bio-concentration factors in Table 4. In general, the BCF of the four elements were small, which means that the metals in the soil were unable to be transferred to the asparagus. The BCF of cadmium and mercury were greater than those of lead and arsenic. In spring and summer, cadmium bio-concentration factor was higher than that of mercury, but it was the opposite in autumn. There was little difference in the BCF of lead and arsenic in the three seasons.

The enrichment capacity of lead was strongest in the east in spring and autumn, and there was no obvious regional difference in lead enrichment capacity in summer. The accumulation capacity of cadmium varied greatly between seasons and regions: the strongest is in the west in spring, the middle in summer, and the east in autumn. Mercury had the strongest accumulation ability in the central part in the three seasons, while arsenic had the strongest in the west in summer and autumn, and there was little regional difference in spring. The enrichment capacity of the four metals was similar in the three regions, all of which were Cd > Hg > As > Pb. In the eastern region, the four types of elements had the strongest accumulation ability in autumn. The enrichment capacity of other metals was strongest in autumn, except for cadmium (summer) in the central region. In the western region, the enrichment capacity of lead was strongest in summer, while that of cadmium, mercury, and arsenic was strongest in autumn.

Taking the region as the classification standard, we found the following phenomena. The enrichment capacity of lead was the strongest in the east, while the regional difference of cadmium was small. Mercury had the strongest enrichment capacity in the central region, and there was little difference of BCF between the east and west. The enrichment capacity of arsenic was the strongest in the west, but the difference was small. It was found that, except for cadmium, the strongest accumulation capacity of the other three elements was in autumn when taking season as the classification standard. The enrichment capacity of cadmium was strongest in summer, but there was little seasonal difference between summer and autumn. The enrichment coefficient of arsenic had significant seasonal differences (*p* < 0.05), which was consistent with our analysis of these metal content in asparagus. Correlation analysis showed that the BCF of cadmium was negatively correlated with soil pH, and the correlation was extremely significant (*p* < 0.01), which was possibly due to soil pH affecting the bioavailability, mobility, and availability of the metal cadmium in the soil-crop system [51].

### 3.3. Single Factor and Comprehensive Pollution Index

**Figure 5:**
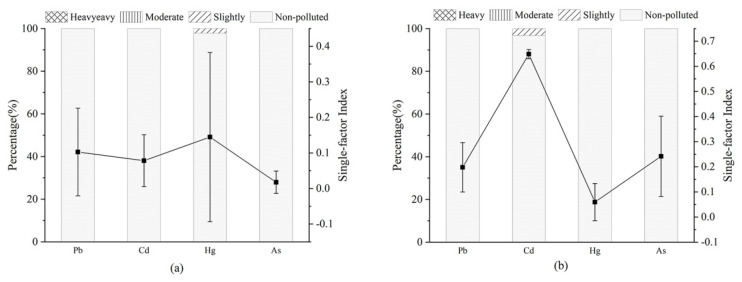
Single-factor pollution level of asparagus (**a**) and soil (**b**) in Chongming Island.

The single-factor pollution levels of the four metals in asparagus and most of soil samples were safe, as shown in Figure 5, and the SFPI of the soil was higher than that of asparagus. The asparagus had the highest single-factor pollution level of mercury and the lowest of arsenic, while the soil had the highest of cadmium and the lowest of mercury, which can also indicate that there was no obvious correlation between metals in the soil and those in asparagus. The SFPI of asparagus in autumn was the highest among the three seasons, and the SFPI of soil in summer was the lowest. For asparagus, the higher SFPI in the eastern and central regions was lead, and the higher SFPI in the western region was mercury, while for soil, the SFPI of cadmium in the soil was higher in each region.

The Nemerow comprehensive pollution levels of asparagus and soil sampled in spring and summer were all safe (Table 5). The NPI of the eastern asparagus in spring was higher than that of other regions, and the NPI of soil in the west was higher in each season. When sampling in autumn, we found that only the comprehensive pollution level of the central soil was slightly polluted, and the level of the rest was safe. In the east and west, the comprehensive pollution levels of asparagus and soil were safe in the three seasons, and the NPI of asparagus in autumn was slightly higher than that in the other two seasons. In the middle part of the region, asparagus was slightly polluted in autumn, and the pollution levels of asparagus and soil in other seasons were safe. However, the NPI of asparagus in autumn was slightly higher, which was the same with the other two regions.

Taking the region as the classification index, the results showed that the Nemerow pollution level of asparagus and soil was safe. The NPI of asparagus in the central area was slightly higher than that of other areas, while the NPI of the western soil was higher. Analyzing samples from three different seasons, we discovered that the Nemerow pollution level of asparagus in autumn was still clean, the NPI of spring and summer was not much different, and they were all safe. The pollution level of soil was safe in all seasons, among which the highest NPI was in spring.

### 3.4. Geo-Accumulation Index of Chongming Asparagus Planting Soil

**Figure 6:**
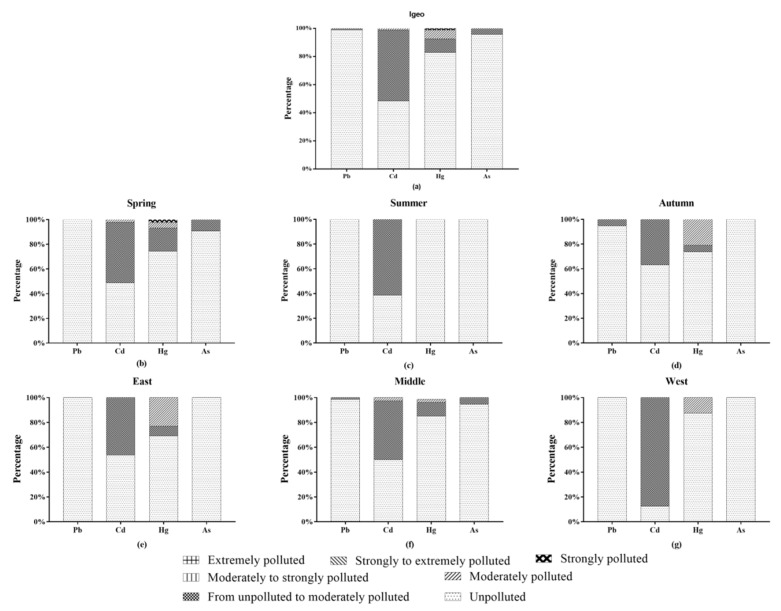
The results of I_geo_ of metals in the soil: (**a**) all samples, (**b**–**d**) different seasons, (**e**–**g**) different regions.

The geological accumulation indexes of lead and arsenic in the three regions were relatively low (Figure 6). Only a few samples in the central region were unpolluted to moderately polluted (1.35% for lead and 5.41% for arsenic), and the rest were unpolluted. Compared with these two metals, the I_geo_ of cadmium and mercury was higher. For cadmium, half of the samples in the three regions were unpolluted to moderately polluted, and a few samples were moderately polluted. The I_geo_ of mercury in the three regions was mostly unpolluted, and there were also some samples that were moderately polluted. The I_geo_ of the four metals had little regional differences, while seasonal differences were greater. The pollution degree of I_geo_ was the lowest in summer, and only some samples’ pollution level of cadmium was unpolluted to moderately polluted. All samples in spring and summer were unpolluted in terms of lead, while a small number samples (4.76%) in autumn were unpolluted to moderately polluted. A few samples (9.3%) of arsenic in spring were unpolluted to moderately polluted, while arsenic in summer and autumn samples were all unpolluted. Mercury in the samples showed similar pollution degrees in spring and autumn.

In general, cadmium was the most seriously polluted among the four metals. No matter the season or region, the I_geo_ of cadmium was higher, and the proportion of samples with heavy pollution degree was also higher than that of other metals. This is consistent with the pollution situation of Chalan Beel Wetland studied by Mohammad Abdus Salam et al. [54]. R. Biddau et al. [55] found that the uptake of Cd in asparagus was higher than that of other toxic elements, hence it is necessary to pay attention to the Cd content in the soil. Lead was the least polluted of these metals, and only a very few samples (1.05%) were unpolluted to moderately polluted, while the rest were unpolluted. The pollution condition of mercury and arsenic was similar but slightly different, and most samples were unpolluted by them. In the rest of the samples, mercury pollution degree was unpolluted to moderate polluted (9.47%), moderately polluted (6.32%), and severely polluted (1.05%), while the arsenic pollution degree was unpolluted to moderately polluted (4.21%).

### 3.5. Human Health Risk Assessment of Chongming Asparagus

The health risk model recommended by USEPA divided the evaluation indicators into carcinogenic risk and non-carcinogenic risk, which was used for the risk assessment of carcinogens and non-carcinogens.

**Figure 7:**
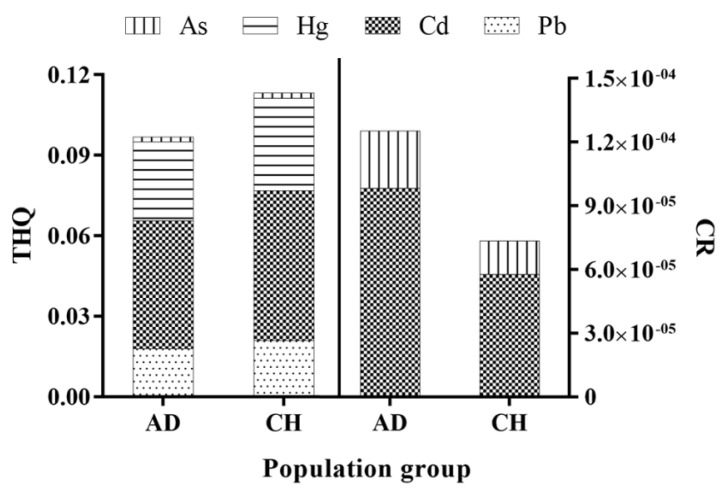
The results of human health risks (AD: adult, CH: children).

In the study area, human health risks for adults and children under the oral exposure pathway were calculated and illustrated in Figure 7 on the basis of the above health risk assessment model and parameters. In this exposure route, the HQ of each metal and the HI of the four metals were less than 1 for both children and adults, indicating that the four elements in asparagus in this area will not cause any significant non-carcinogenic health risk. Li et al. found that the human non-carcinogenic risk of these metals in Shanghai industrial zone was negligible [56]. The health risks were related to dietary intake. Andrea Ariano’s [57] research showed that although the average concentrations of lead in muscle samples of octopus were above the maximum concentration level of 0.3 µg/g, it still had no effect on human health due to low consumption. The non-carcinogenic health risks caused by metals in asparagus were in the order of cadmium > mercury > lead > arsenic. Specifically, cadmium had the highest non-carcinogenic risk, which was about twice or more than that of other three metals. Comparing the THQ of adults and children, we found that the latter was 1.17 times higher than the former, which may have mainly been due to the lower weight of children. Under the same environmental conditions, children face higher non-carcinogenic health risk than adults, which is consistent with the study of Tian et al. [6].

Among the four elements, the carcinogenic slope factors of lead and mercury were not found in the literature, only the carcinogenic risk of cadmium and arsenic to humans was studied ultimately. The carcinogenic health risk index of cadmium and arsenic ranged from 1.0 × 10^–6^ to 1.0 × 10^–4^, which means that the carcinogenic risk was acceptable, although the carcinogenic risk of cadmium was over three times higher than that of arsenic. The carcinogenic risk of adults was 3.6 times higher than that of children, possibly due to their higher intake and longer exposure.

## 4. Conclusions

We performed metal content determination and risk analysis of asparagus, soil, and water in Chongming Island. The contents of four metals in asparagus in Chongming Island had a common rule of Pb > As > Cd > Hg and were all lower than Chinese national limit standards. There were obvious seasonal differences of arsenic content, while all four heavy metals we studied had no significant regional differences of contents and BCF in asparagus and soil in Chongming Island. The BCF of these elements in asparagus were all less than 0.5, indicating that they could not be accumulated from soil to plants during growth and the enrichment ability of cadmium was the strongest.

Three indices were used to assess metal contamination in asparagus and soil of Chongming. In view of the results of single-factor pollution index, Nemerow pollution index, and geological accumulation index, we found cadmium pollution to be highest in over one-half of soil samples, and 51.62% of them were slightly polluted. It is practical to cultivate plants that are hard or adversarial to accumulate cadmium in Chongming Island. According to human health risk assessment, there was no obvious non-carcinogenic risk from eating asparagus, and the carcinogenic risk of cadmium and arsenic was also acceptable. Therefore, asparagus in Chongming area is safe for consumers.

## Figures and Tables

**Table 1:** The standard of single-factor pollution index and Nemerow pollution index.

Single-Factor Pollution Index		Nemerow Pollution Index	
Index	Pollution Level	NPI	Pollution Level
SFPI ≤ 1	Non-polluted	NPI ≤ 0.7	Clean (safe)
1 < SFPI ≤ 2	Slightly	0.7 < NPI ≤ 1	Still clean (cordon)
2 < SFPI ≤ 3	Moderate	1 < NPI ≤ 2	Slightly
SFPI > 5	Heavy	2 < NPI ≤ 3	Moderate
		NPI > 3	Heavy

**Table 2:** The criteria of geo-accumulation index.

I_geo_	Pollution Category
I_geo_ ≤ 0	Unpolluted
0 < I_geo_ ≤ 1	From unpolluted to moderately polluted
1 < I_geo_ ≤ 2	Moderately polluted
2 < I_geo_ ≤ 3	Moderately to strongly polluted
3 < I_geo_ ≤ 4	Strongly polluted
4 < I_geo_ ≤ 5	Strongly to extremely polluted
I_geo_ > 5	Extremely polluted

**Table 3:** Parameters related to metal health risks.

Parameter	Definition	Value	Indicator Source
E_f_ (d/a)	Exposure frequency	350	[39]
E_D_ (a)	Exposure period	Adult: 24	[38]
		Child: 6	
E_IR_ (mg/d)	Ingestion rate	Adult: 100	[38]
		Child: 200	
c (mg/kg)	Concentration of metal	This study	This study
R_f_D (mg·kg^−1^·d^−1^)	Reference dose	Pb: 0.0035	Cd, Pb, As: [40] Hg: [41]
		Cd: 0.001	
		Hg: 0.0003	
		As: 0.03	
W_AB_ (kg)	Body weight	Adult: 56.8	[38]
		Child: 15.9	
T_A_-non-carcinogenic (days)	Total time of non-cancer risk exposure	Adult: 24 × 365	[42]
		Child: 6 × 365	
T_A_-carcinogenic (days)	Total time of cancer risk exposure	Adult: 26,280	[38]
		Child: 26,280	
SF(μg·g^−1^·day^−1^)	Slope factor	Cd: 6.1, As: 1.5	[38]

**Table 4:** BCF of metals in different seasons and regions.

		Pb	Cd	Hg	As
Spring	East	0.0011 ± 0.0010	0.0254 ± 0.0203	0.0034 ± 0.0020	0.0003 ± 0.0001
	West	0.0002 ± 0.0001	0.0313 ± 0.0149	0.0026 ± 0.0007	0.0003 ± 0.0000
	Middle	0.0003 ± 0.0002	0.0183 ± 0.0178	0.0057 ± 0.0105	0.0003 ± 0.0001
Summer	East	0.0004 ± 0.0002	0.0587 ± 0.0113	0.0062 ± 0.0009	0.0021 ± 0.0008
	West	0.0007 ± 0.0000	0.0499 ± 0.0124	0.0078 ± 0.0031	0.0027 ± 0.0012
	Middle	0.0007 ± 0.0005	0.0680 ± 0.0708	0.0082 ± 0.0036	0.0011 ± 0.0006
Autumn	East	0.0096 ± 0.0093	0.0592 ± 0.0182	0.0152 ± 0.0017	0.0080 ± 0.0008
	West	0.0003 ± 0.0003	0.0574 ± 0.0082	0.0139 ± 0.0067	0.0110 ± 0.0084
	Middle	0.0032 ± 0.0094	0.0591 ± 0.0396	0.1055 ± 0.1314	0.0033 ± 0.0030

**Table 5:** Nemerow pollution index of asparagus and soil.

		Asparagus		Soil	
Season	Region	Pollution Index	Pollution Level	Pollution Index	Pollution Level
Spring	East	0.2208 ± 0.2027	Clean	0.4846 ± 0.099	Clean
	Middle	0.0915 ± 0.0916	Clean	0.5413 ± 0.1567	Clean
	West	0.0632 ± 0.0252	Clean	0.5918 ± 0.0803	Clean
Summer	East	0.1089 ± 0.0145	Clean	0.5098 ± 0.0865	Clean
	Middle	0.1211 ± 0.0759	Clean	0.4847 ± 0.1136	Clean
	West	0.1108 ± 0.0037	Clean	0.5412 ± 0.0301	Clean
Autumn	East	0.4392 ± 0.0453	Clean	0.4542 ± 0.1112	Clean
	Middle	1.0395 ± 2.0091	Slightly	0.5275 ± 0.3349	Clean
	West	0.4718 ± 0.3637	Clean	0.5368 ± 0.0016	Clean

## Data Availability

The datasets used and/or analyzed during the current study are available from the corresponding author on request.
